# Supplementary material for: Development of clinical prediction rule for the requirement of endoscopic papillary large balloon dilation (EPLBD) on endoscopic CBD stone clearance
Source: PLoS One. 2023 Mar 16;18(3):e0282899. doi: 10.1371/journal.pone.0282899 (PMC10019717; doi:10.1371/journal.pone.0282899)
Supplement: Attachment — Submitted filename: Response to Reviewers.docx [file pone.0282899.s001.docx]

Dear academic Editor

Thank you for giving me the opportunity to submit a revised draft of my manuscript titled

"Development of clinical prediction rule for the requirement of endoscopic large papillary balloon dilation (EPLBD) on endoscopic CBD stone clearance" to PLOS ONE. I appreciate the time and effort that you and the reviewers have dedicated to providing your valuable feedback on my manuscript. I am grateful to the reviewers for their insightful comments on my paper. I have been able to incorporate changes to reflect most of the suggestions provided by the reviewers. I have highlighted the changes within the manuscript. Here is a point-by-point response to the comments and concerns.

Journal Requirements:

*1. Please ensure that your manuscript meets PLOS ONE's style requirements, including those for file naming……….*

**Reply:** We have revised the manuscript to meet the style requirements of PLOS ONE for the main body, title, authors, and affiliations.

*2.*  Thank you for stating the following in the Acknowledgments Section of your manuscript: 
"This research work was partially supported by Chiang Mai University, Chiang Mai, Thailand."

We note that you have provided funding information that is not currently declared in your Funding Statement. However, funding information should not appear in the Acknowledgments section or other areas of your manuscript. We will only publish funding information present in the Funding Statement section of the online submission form. 
Please remove any funding-related text from the manuscript and let us know how you would like to update your Funding Statement. Currently, your Funding Statement reads as follows: 
"The funders had no role in study design, data collection and analysis, decision to publish, or preparation of the manuscript."

Please provide an amended statement that declares *all* the funding or sources of support (whether external or internal to your organization) received during this study, as detailed online in our guide for authors at http://journals.plos.org/plosone/s/submit-now.  Please also include the statement “There was no additional external funding received for this study.” in your updated Funding Statement.

Please include your amended statements within your cover letter; we will change the online submission form on your behalf.

**Reply:** We removed the funding-related text "Chiang Mai University partially supported this research work, Chiang Mai, Thailand," from our manuscript. And we will add the funding in the funding statement.

*3. Your ethics statement should only appear in the Methods section of your manuscript. If your ethics statement is written in any section besides the Methods, please delete it from any other section.*

**Reply:** The ethics statement is only in the Materials and methods section of our manuscript. So, the ethical information is only written here on page 5, line 4 of the first paragraph.

*4. Please include a caption for figure 5.*

**Reply:** The caption for figure 5 appears on page 22 of our manuscript

"**Fig 5.** **Comparison between the area under the receiver operating characteristic curve of DCSS and DSAS.**

The Difficult CBD stone score (DCSS) model (blue line) and the difficulty scoring and assistance system (DSAS) for endoscopic treatment of common bile duct (CBD) stones (red line)".

*5. Please ensure that you refer to Figure 5 in your text as, if accepted, production will need this reference to link the reader to the figure.*

**Reply:** Figure 5 was referred in our text on page 12, line 6 of the second paragraph.

Additional Editor Comments:

Response to Reviewers

Reviewer 1

**Reviewer:** *1.1. The author did not mention if they included only adult patients*

**Reply:** This study included only adult patients. Thank you for pointing this out. I agree with this comment. Therefore, I have clearly described the purpose of this study on page 5, line 1 of paragraph 2.

**Reviewer:** *1.2. Were the procedures (ERCPs) done under MAC or general IV anesthesia?*

**Reply:** Agree. I have, accordingly, clarified to emphasize this point. All patients were done procedures under general IV sedation. Therefore, I have clearly described this on page 6, line 6 of paragraph 1.

**Reviewer**: *1.3. Which was the number and experience of the endoscopists?*

**Reply:** In this study, experienced endoscopists performed over 300 ERCP procedures. Therefore, I have described in detail on page 5, line 2 of paragraph 2.

**Reviewer:** *1.4. One-third of the patients who required EPLBD have scores <5….this seems to be a very high proportion.*

**Reply :** You have raised an important point here. However, We selected a cut point value at 5 because of high specificity (94.74%) and sensitivity (72.15%), allowing for the inclusion of individuals with more difficult CBD stones. In addition, at this cut point, It early realizes that bile duct stones are difficult to treat and need a dilation or to make the stones smaller. Therefore, I have clearly described on page 12, line 3 of paragraph 1.

**Reviewer:** *1.5. Maybe my most important question is the following: this new score that the authors are proposing needs information obtained through/during the ERCP procedure. After you get it you calculate the score (ex. To calculate the CBD angle, you need to measure the angle formed by the CBD between 1 cm below the bifurcation and 1 cm above the papilla, the distance between the ampullary orifice to the distal CBD angulation point, the longitudinal diameter and transverse diameter of the stone at the point of maximum diameter, etc)….so which is the advantage in doing all this (with the time that takes to do it) vs to do a swept with the extraction balloon (and if you see is not possible, go directly to EPLBD or refer the patient).*

**Reply:** You have raised an important point here. However, in the case of our study, this simple scoring system is easy to use by estimating the diameter of the CBD stone, distal CBD diameter, and distal CBD arm compared to the actual diameter of the duodenoscope by the rule of three calculators based on proportion with the short time to do it during ERCP. The stone shape was observed directly on cholangiography. Patients who scored more than 5 indicated that it was necessary to gain CBD access or reduce the size of the stone before stone clearance, which aids in determining the following procedure and choice of sedation because it prolongs operations and aids in deciding whether to proceed with the procedure or refer the patient based on feasibility in that hospital.

**Reviewer:** *1.6. PLOS authors have the option to publish the peer review history of their article (what does this mean?). If published, this will include your full peer review and any attached files.*

**Reply:** We follow the guideline for publication of PLOS one. If accepted for publication, published version of manuscript will be followed with PLOS guideline.

We look forward to hearing from you in due time regarding our submission and to responding to

any further questions and comments you may have.

Sincerely,

Chote Wongkanong M.D.

Apichat Tantraworasin M.D., Ph.D.
